# Suicide trends in Norway during the first year of the COVID-19 pandemic: A register-based cohort study

**DOI:** 10.1192/j.eurpsy.2022.17

**Published:** 2022-04-19

**Authors:** K. Stene-Larsen, G. Raknes, B. Engdahl, P. Qin, L. Mehlum, M. S. Strøm, A. Reneflot

**Affiliations:** 1 Department of Mental Health and Suicide, National Institute of Public Health, Oslo, Norway; 2 Raknes Research, Ulset, Norway; 3 Department of Physical Health and Ageing, National Institute of Public Health, Oslo, Norway; 4 National Centre for Suicide Research and Prevention, Institute of Clinical Medicine, University of Oslo, Oslo, Norway; 5 Department of Health Registry Research and Development, National Institute of Public Health, Bergen, Norway

**Keywords:** COVID-19, pandemic, suicide

## Abstract

**Background:**

There is a concern that the COVID-19 pandemic will lead to an increase in suicides. Several reports from the first months of the pandemic showed no increase in suicide rates while studies with longer observation times report contrasting results. In this study, we explore the suicide rates in Norway during the first year of the pandemic for the total population as well as for relevant subgroups such as sex, age, geographical areas, and pandemic phases.

**Methods:**

This is a cohort study covering the entire Norwegian population between 2010 and 2020. The main outcome was age-standardized suicide rates (per 100,000 inhabitants) in 2020 according to the Norwegian Cause of Death Registry. This was compared with 95% prediction intervals (95% PI) based on the suicide rates between 2010 and 2019.

**Results:**

In 2020, there were 639 suicides in Norway corresponding to a rate of 12.1 per 100,000 (95% PI 10.2–14.4). There were no significant deviations from the predicted values for suicides in 2020 when analyzing age, sex, pandemic phase, or geographical area separately. We observed a trend toward a lower than predicted suicide rate among females (6.5, 95% PI 6.0–9.2), and during the two COVID-19 outbreak phases in 2020 (2.8, 95% PI 2.3–4.3 and 2.8, 95% CI 2.3–4.3).

**Conclusion:**

There is no indication that the COVID-19 pandemic led to an increase in suicide rates in Norway in 2020.

## Introduction

There is a concern that the COVID-19 pandemic will have a profound impact on the population’s mental health and in turn also on suicide risk [1]. The duration and extent of government implemented measures, such as physical distancing and redistribution of mental health care resources that could potentially increase isolation, economic recession and reduced access to mental health services is especially concerning [2–7]. Previous research on suicide risk during pandemics is scarce but indicates that the association is complex as some studies have found an increase in suicides [8,9] whereas others have found an initial reduction followed by an increase later on [10,11].

With regard to the COVID-19 pandemic, reports from the first months of the pandemic have shown stable suicide rates both in Norway and in the USA [12,13]. Other countries have reported similar trends. For instance, a report using monthly suicide figures from 21 high-income or upper-middle-income countries found no evidence of an increase in suicides in any of these countries as of July 2020 [14]. Similar results have also been found in Queensland, Australia, and Greece during the first 6 months of the pandemic [15,16]. In Maryland, the observations from the first 6 months of the pandemic have shown a decrease in suicides among white Americans and an increase in suicides among other ethnic groups [17].

Two studies examined suicide rates until October 2020 [18,19]. The first of these presented suicide rates in a German city and found that suicide rates were lower during phases with the most restrictions [19]. The second study, from a UK population, used real-time surveillance data of suspected suicides and analyzed suicide trends from the first lockdown in March 2020 to the second lockdown in October 2020 [18]. The authors found no evidence of an increase in suicides despite several reports indicating increased mental health distress in the population.

Studies that report on suicide rates from the complete first year of the pandemic have shown contrasting findings. Some have found stable or decreased suicide rates [20,21] whereas others have found variable patterns of suicide risk varying either by time or by specific subgroups of the population [22,23]. For instance, in a study of suicide trends in the Japanese population suicide rates were found to have dropped significantly in the early months of the pandemic followed by a sharp increase in the second half of 2020 [23]. The increase in suicide rates was primarily observed among young adolescents and women. In a study from Taiwan, covering the first 12 months of the pandemic an increase in suicide rates was found among those aged 25 years or younger and a decrease among those aged 25–64 [22]. For those aged 65 years and above an initial decrease was observed in the first parts of the pandemic followed by an increase in suicides in the later phases. In addition to these studies on suicide trends, several reports have demonstrated reductions in rates of self-harm [24,25] and suicide attempt [26] during the pandemic.

To gain further insight into the dynamics of suicide risk during pandemics, studies from differing cultural contexts, with varying degrees of how hard the pandemic has hit and with sufficient observation time are warranted. Increased knowledge regarding age-specific suicide risk is needed as certain age categories such as the elderly [27–29] and young adults [22,30] could be especially vulnerable. As economic downturns are expected during the pandemic, a factor potentially affecting males more than females in most countries through labor market marginalization effects, a focus on suicide risk among males is also important [31].

## Aims

In this study, we aim to explore changes in suicide rates during the first year of the COVID-19 pandemic in the Norwegian population. We present suicide rates by age and sex from the first year of the COVID-19 pandemic in Norway and compare these with the suicide rates during 10 pre-pandemic reference years (2010–2019). In addition, we present stratified analyses for the capital region of Norway, which had continuously high infection rates, by pandemic phases related to restrictions throughout 2020, and by age and sex with particular foci on suicide risk among the elderly, young adults, and on men.

## Methods

We conducted a nationwide-register-based cohort study with data from the Norwegian Cause of Death Register including all registered deaths by suicide (ICD-10 codes X60-X84 and Y87.0) from January 1, 2010 to December 31, 2020. The data comprise all Norwegian citizens including those who died abroad. Foreign citizens who die in Norway are listed in the register, but are not part of the official statistics nor the analyses presented in this article.

Age-standardized suicide rates (per 100,000 inhabitants) were calculated using the European Standard Population of years 2013 [32]. For each year we categorized data into the following seasonal periods: January–February, March–May, June–September, and October–December. This allowed us to analyze possible changes in suicide occurrence during four specific phases of the pandemic, that is, the pre-pandemic period (January–February), the first wave of outbreak (March –May), the intermediate period (June–September), and the second wave of the outbreak period (October–December), roughly classified according to the outbreak status and prevention measures throughout the year 2020 in Norway [33,34].

In addition, we calculated age-standardized suicide rates by the following strata: Sex and the Norwegian capital region (yes or no). Separate analyses on the age groups (15–24 years, 25–44 years, 45–64 years or above 65 years) were also performed. The Capital region consists of the municipality of Oslo and 19 surrounding municipalities from Viken county with a population of 1,338,600, accounting for 24.9% of the national population in 2020. In this geographic area, the SARS-COV2-transmission rate was continuously high and restrictions on mobility were thus stricter and lasted longer than in the rest of the country. The years 2010–2019 were used as a comparison, for both whole years and corresponding months, and for subgroup analyses.

Most doctors who confirmed a death reported the cause of death to the Norwegian cause of death register and the National population register by filling in the international death certificate (1993 version) which was sent by post-mail. An increasing number of death certificates were submitted using a new digital tool that was being introduced in Norway during the observation period [35]. The final ICD-10 underlying cause of death in the registry based on the death certificate is determined by an automated coding system (IRIS) that is based on the Automatic Classification of Medical Entry (ACME) software [36]. For all unnatural deaths (including suicides), a manual evaluation by trained staff in the Norwegian Cause of Death Registry is applied. Most suicides (~80%) are routinely confirmed by autopsies.

### Statistical analyses

Age-standardized mortality rates were computed by the direct standardization method, using 5-year age strata and the European Standard Population of years 2013 [32]. Information on “at risk” population (mid-year population) was obtained from the Statistics Norway [37].

For the primary analyses, we used a negative binomial regression model to estimate the standardized suicide rate and the 95% prediction intervals for the total population in 2020 based on data from the complete reference period 2010–2019. Population size was included as an offset term in the model. Due to lower levels of over-dispersion in the data for sex, age, pandemic phase, and geographical area we used Poisson regression models with population size included as an offset term to model the suicide rates for 2020 in these analyses. The 95% Prediction Intervals were estimated using bootstrapping.

For the four phases of the pandemic, the regression line and 95% prediction intervals were based on the corresponding months in the comparison years (2010–2019). For instance, the observed suicide rate in the period of January 2020 to February 2020 was compared with the predicted rate based on the data from January to February during the 10 comparison years (2010–2019), the suicide rate in the period of March 2020 to May 2020 was compared to the suicide rate in March–May 2010–2019 and so on. This accounts for both time trends over the total observation period and potential seasonal effects. The data on the pandemic phases were tested for autocorrelation using the Watson Durbin test. We did not find evidence of autocorrelation that needed to be adjusted for in the analyses.

All models were based on the total number of suicides in the reference years with some exceptions regarding the age-specific analyses. In the age-stratified analyses, we did not calculate suicide rates for the youngest age category (0–14 years) due to very few suicides in this age category making it difficult to conduct statistically sound analyses. Also, in the analyses combining suicide, age, and pandemic phase we aggregated the age categories 0–14 years and 15–25 years in order to maintain anonymity in the data. For all analyses, observed suicide rates falling outside of the projected 95% confidence interval in 2020 were considered statistically significant deviations.

### Ethics and data availability

The underlying data aggregates used in this study are found in the Supplementary Material, but can also be provided on request to the Norwegian cause of Death Registry at (www.helsedata.no). The data used were either aggregated or anonymous register data. Approval from the Regional Committee for research is not required using aggregated or anonymous registered data.

## Results

Table 1 shows baseline data for suicide deaths for 2020 and the reference period (2010–2019). A total of 6,549 suicides were registered, of which 639 in 2020. The median age of the suicides was 47 years in 2020, similar to the mean age of reference years. The proportion of female suicides was slightly lower in 2020 (26.9%) than the average of the reference years (29.8%).

**Table 1.** Background characteristics of suicide deaths in Norway from 2010 to 2019 and in 2020.

N	2010–2019		2020	
	5,910		639	
Mean age (±SD)	46.7	(±17.9)	47.2	(±18.5)
Median age (IQR)	46	(32–59)	47	(32–62)
*N* (%) females	1,763	(29.8)	172	(26.9)
*N* (%) age 0–14	34	(0.6)	5	(0.8)
*N* (%) age 15–24	724	(12.3)	72	(11.3)
*N* (%) age 25–44	1,970	(33.3)	221	(34.6)
*N* (%) age 45–64	2,174	(36.8)	208	(32.6)
*N* (%) age 65+	1,008	(17.1)	133	(20.8)
*N* (%) Oslo area	1,384	(23.4)	143	(22.4)

In Table 2, the age-standardized suicide rates are compared with predictions based on 2010–2019 data. The Supplementary Material provides detailed information on the number of suicides and suicide rates for each year of the observation period.

**Table 2.** Age-standardized suicide rates (per 100,000) in 2020 compared with predicted value with 95% prediction interval from the regression models based on observational data from 2010 to 2019.

		Suicide rate (per 100,000)		
		Predicted 2020	Observed 2020	95% prediction interval
Total population		12.3	12.1	10.2–14.4
Sex	Male	17.1	17.6	14.8–19.5
	Female	7.6	6.5	6.0–9.2
Geographic area	Capitol region	12.3	11.3	10.3–14.4
	Norway (Capitol region excluded)	12.6	12.4	10.5–14.6
Age groups	0–14 years^a^	—	—	—
	15–24 years	11.3	11.0	6.7–16.0
	25–44 years	14.5	15.2	9.1–20.0
	45–64 years	17.2	14.9	11.3–23.1
	65+ years	13.2	13.9	8.7–17.6
By months	January–February	1.9	2.2	1.3–2.6
	March–May	3.3	2.8	2.3–4.3
	June–September	3.9	4.2	2.8–4.9
	October–December	3.3	2.8	2.3–4.2

a Statistical analyzes on the age category 0–14 years were not included due to very few suicides in this age category.

**Figure 1.**
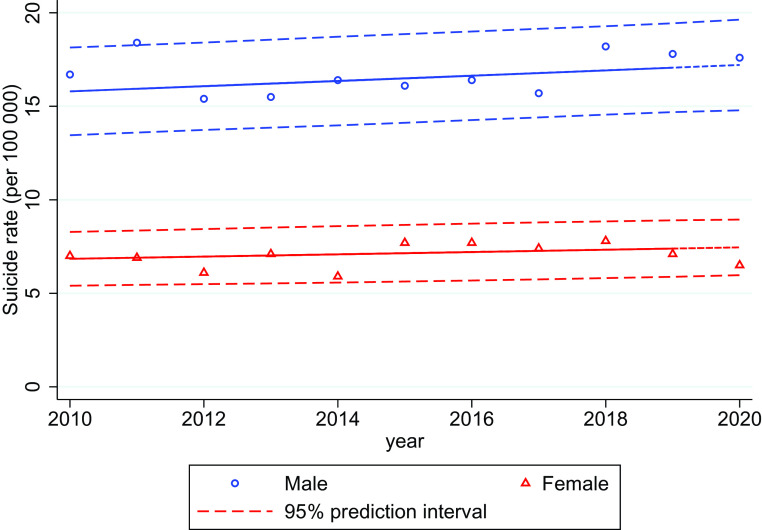
Sex stratified age-standardized suicide rates (per 100,000) 2010–2020 with 95% prediction intervals.

In 2020, the standardized suicide rate in the total population was 12.1 per 100,000 population; this does not deviate from the predicted rate based on 2010–2019 data. The suicide rate for males was close to the predicted value. For females, the 2020 suicide rate was the third lowest since 2010 but still within the 95% prediction interval (see Figure 1).

For all age groups, the suicide rates were all within the 95% prediction intervals, meaning that there were no statistically significant deviations from trends between 2010 and 2019. The suicide rate among those aged 45–64 was the lowest observed since 2010 but still within the interval and hence not significantly lower than expected. The suicide rate in the oldest age group (65+) was the second highest since 2010, but still within the 95% prediction interval.

**Figure 2.**
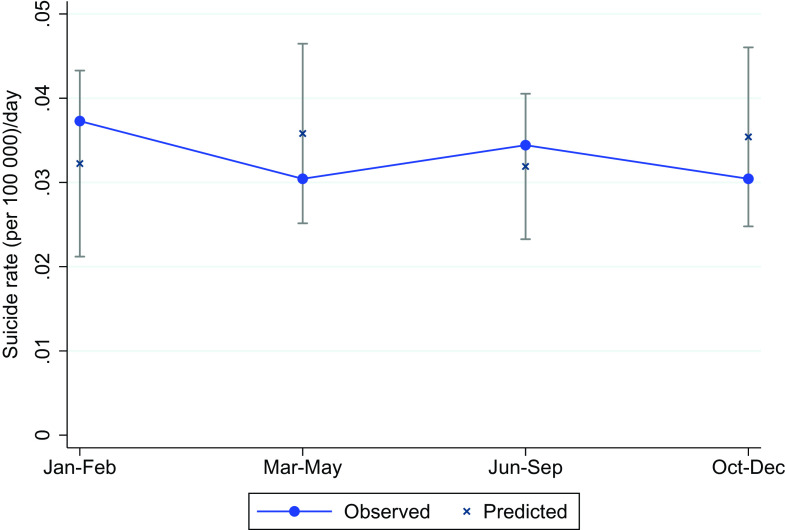
Average age-standardized death rate (per 100,000) per day in four phases of 2020, compared with predicted age-standardized death rates with 95% prediction intervals based on corresponding months of 2010–2019.

For all four pandemic phases, the suicide rates were all within the 95% prediction intervals and hence did not deviate from the trends between 2010 and 2019 (Table 2). Even though the suicide rates were all within the 95% Prediction intervals we did reveal a pattern where the observed suicide rates were lower than the predicted rates during the outbreak periods (March–May) and (October–December) and higher than predicted in the pre-pandemic phase (January–February) and in the intermediate phase (June–September) (Figure 2).

**Figure 3.**
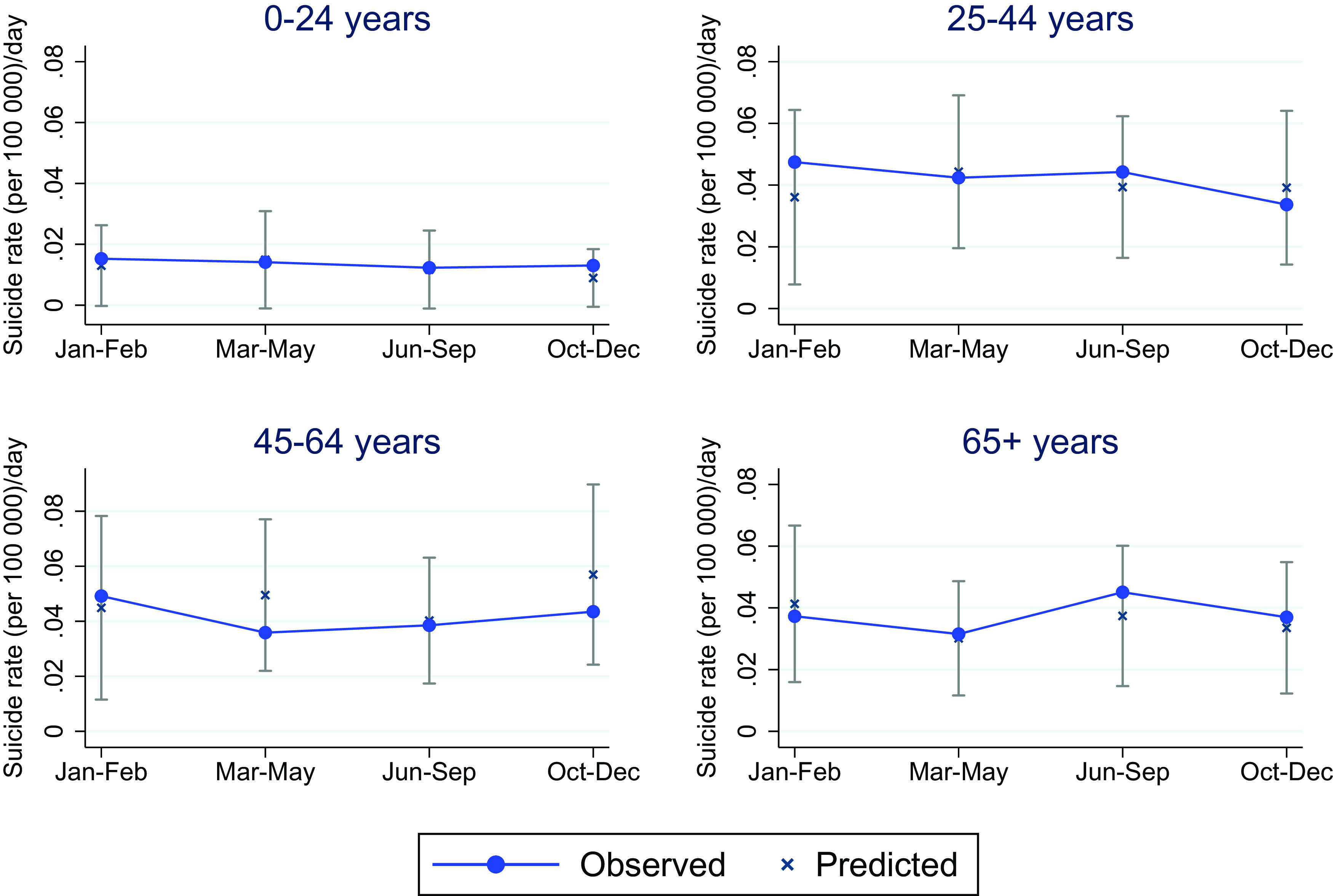
Age stratified standardized suicide rate (per 100,000) per day in the four pandemic phases of 2020, compared with predicted age-stratified suicide rates with 95% prediction intervals based on the corresponding phases of 2010–2019.

When stratifying the pandemic phases into age groups (Figure 3), the suicide rate for those aged 45–64 years was around 20% lower than predicted in both outbreak phases (March–May) (October–December). For the remaining age groups, the observed suicide rates were in line with the predicted rate in all four phases of the pandemic.

**Figure 4.**
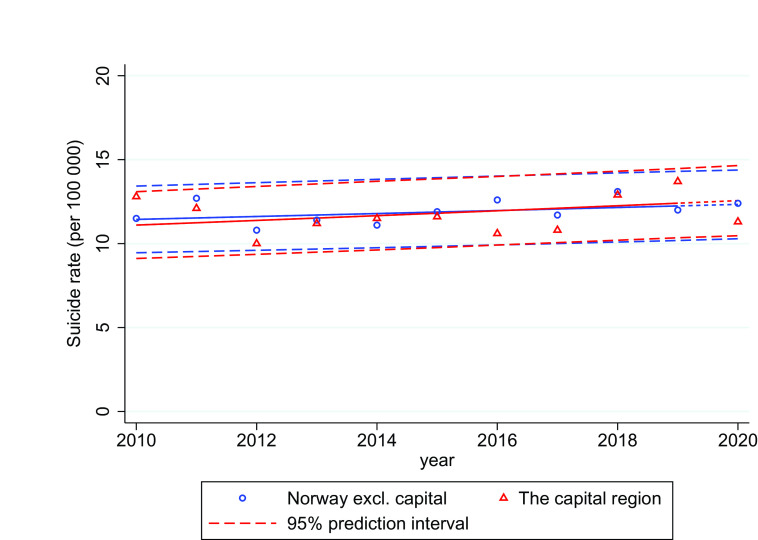
Age-standardized suicide rates (per 100,000) in the Capitol region and rest of Norway 2010–2020 with 95% prediction interval.

Figure 4 shows the results from the regression model for the Capitol region compared to the rest of the country. For the Capitol region there were slightly fewer observed suicides in 2020 than in the comparison years, but well within the 95% prediction intervals. For the rest of the country, the suicide trends for 2020 were stable and follow the prediction line.

## Discussion

This study provides an overview of suicide trends in Norway during the first year of the COVID-19 pandemic. Although we found no statistically significant changes in suicide rates in 2020 compared to the previous decade, we discovered several trends that we believe merit considerable interest. First, we found no evidence of an increase in suicides in the Norwegian population as a whole. Second, we found the lowest and the third lowest suicide rates in the last decade among those aged 45–64 and women respectively. Third, we found the second highest suicide rate over the last decade among those 65 years and older. Fourth, we found that the suicide rates were lowest during the most profound outbreak periods with strict mobility restrictions and higher during periods of less restrictions. Finally, we found no major deviations in suicide rates in the capital region despite that this area was under a heavy burden of a combination of high infection rates and mobility restrictions and regulations throughout the year.

The stable suicide rates observed in the total population during 2020 stand in contrast to the warnings of a possible increase in suicides in the early phases of the pandemic [2,30]. Our findings also stand in contrast to the increase in suicide rates observed during the influenza pandemic in 1918–1920 [8,9]. On this background the stable suicide rates that we observed in this observational study were surprising, but on the other hand, our findings correspond well with observations from other countries that have reported either a reduction in suicides or stable suicide rates during the early phases of the COVID-19 pandemic [14].

The association between virus pandemics and suicide risk is complex. Social cohesion or the degree of unity in the population is an example of a protective factor that has been observed in the COVID-19 pandemic [38]. Evidence from natural disasters [39], world wars [40], and modern time wars [41] show a similar pattern with increased social cohesion, reduced suicide rates, and protective effects on multiple detrimental mental health outcomes including suicidal ideation [42]. The trend toward fewer suicides observed among women and middle-aged adults in our study could perhaps be explained by increased social cohesion in these groups. Women and middle-aged adults are groups that often have responsibilities caring for children and the elderly. Having to focus on the need of vulnerable dependents and spending more time with family members during periods of mobility restrictions could potentially increase the feeling of unity and belongingness to the family. Reduced number of alternative social contacts could also have reduced the risk of relationship breakups that are a significant risk factor for suicide, especially among men [43].

Even though wars and virus pandemics share many similarities, they are different in other aspects. With regard to the COVID-19 pandemic, one should also keep in mind that the majority of studies of suicide rates so far have come from high- to middle-income countries [14]. The population resilience in high-income countries could be higher than in low-income countries due to their larger resources to cope with the pandemic. The observed deviation in suicide risk during the influenza pandemic in 1918–1920 [8,9] and the COVID-19 pandemic [14] could perhaps, at least partly, be explained by differences in resources to cope with both the virus and the mental health consequences of pandemics. Another factor is that the overall number of infected and deaths due to COVID-19 in Norway has been very low making comparison with other historic pandemics and suicide rates in other countries during the COVID-19 pandemic complicated. In support of this, a time series analysis of mortality data from 37 high and middle-income countries found that Norway was one of the very few countries that did not experience an increase in premature death and reduction in life expectancy during the first year of the COVID-19 pandemic [44].

A concern during the first phases of the pandemic was that increased suicide risk in the population could follow factors such as social isolation, increased prevalence of mental disorders or deterioration of existing mental disorders, or economic recession [45]. Several findings suggest that the pandemic may not have had as strongly negative effect through these factors as one could fear.

In Norway for instance, a study reported stable rates of loneliness in the general population and only a slight increase among vulnerable groups such as single individuals and older people [46]. Others have even found increased rates of social support during the pandemic [47].

With regard to mental disorders, no increase in any of the common mental disorders in Norway was observed during the first phases of the COVID-19 pandemic [48]. In fact, the authors reported a statistically significant reduction in mental disorders during the first outbreak. Findings from international studies also point in the same direction. Results from a living systematic review and meta analyses of diagnostic data from 33 unique cohort studies found no evidence of an increase in symptoms of either anxiety or depression during the COVID-19 pandemic [49]. This was true for both the general population, for males and females, and across several age groups.

The observed trend of an increase in suicides among those aged over 65 years is in line with findings from previous pandemics [27–29] and from findings from previous studies of the COVID-19 pandemic [22]. Unfortunately, we did not have the statistical power to do further in-depth analyses of the oldest in our study.

The trend of decreased suicide risk during the outbreak periods and higher suicide risk outside the outbreak periods is interesting. First, a similar pattern was reported in a study based on register data from a major German city with lower suicide rates corresponding with periods of strict mobility restrictions [19]. The authors attribute the effect to mobility restrictions preventing people from access to outdoor suicide means such as bridges and trains as well as the fact that increases in the time spent with the family could have increased the likelihood that other family members would detect increased suicide risk among vulnerable individuals.

We found that the suicide rates in the capital region did not differ from the rest of the country during the first year of the pandemic even though this area had continuously high infection rates and almost constant mobility restrictions throughout 2020. A similar trend was also observed in Milan, Italy, a city that had a much higher infection rate than Oslo [20]. Many of the same mechanisms as previously discussed, such as increased social cohesion among Oslo area residents, increased awareness of mental health problems by family members, and restricted access to outdoor means of suicide are potential explanations for this finding.

### Limitations

Our study has some limitations that one must keep in mind when interpreting the findings. First, the relatively small number of suicides registered in Norway each year gives obvious limitations with regard to statistical power, especially in analyses using smaller subgroup strata. Another limitation is that there may be a slight underreporting of suicides from the last months of 2020 due to a delay in information from autopsy confirming causes of death. To best of our knowledge, the potential underreporting is not large enough to influence the main finding of stable suicide rates in 2020.

### Implications for future research

This study indicates that virus pandemics do not necessarily increase suicide risk in the population over the short term. However, as the COVID-19 pandemic is still ongoing, we do not know what the impact will be in the longer term. Future studies that monitor the consequences of the COVID-19 pandemic over several years are warranted. Comparative studies that take into account how hard each country was hit, and the types and amount of preventive measures implemented, and consider cultural and economic differences may provide increased insight into the mechanisms behind the differing patterns of suicide risk observed across countries.

## Conclusion

There is no indication that the COVID-19 pandemic led to an increase in suicides in Norway in 2020. Despite of this, we believe there is a need for continuous monitoring of suicides in the time to come as the impact of a prolonged pandemic on suicide risk is unclear, especially among high-risk populations such as the elderly, children, adolescents, and young adults.

## Data Availability

The underlying data aggregates used in this study are found in the Supplementary Material, but can also be provided on request to the Norwegian Cause of Death Registry at www.helsedata.no.

## Supplementary Materials

To view supplementary material for this article, please visit http://doi.org/10.1192/j.eurpsy.2022.17. click here to view supplementary material
