# Supplementary material for: Suicide trends in Norway during the first year of the COVID-19 pandemic: A register-based cohort study
Source: Eur Psychiatry. 2022 Apr 19;65(1):e26. doi: 10.1192/j.eurpsy.2022.17 (PMC9058441; doi:10.1192/j.eurpsy.2022.17)
Supplement: Supplementary file 1 [file S0924933822000177sup001.docx]

# Supplementary file 1

# Data used for analyses of suicides 2010-2019

## All suicides - sex

### 1.1 Number of suicides, total and in males and females Norway 2010-2020

|  | All | Male | Female |
| --- | --- | --- | --- |
| 2010 | 549 | 384 | 165 |
| 2011 | 601 | 437 | 164 |
| 2012 | 520 | 371 | 149 |
| 2013 | 555 | 382 | 173 |
| 2014 | 551 | 404 | 147 |
| 2015 | 596 | 404 | 192 |
| 2016 | 617 | 421 | 196 |
| 2017 | 595 | 405 | 190 |
| 2018 | 674 | 472 | 202 |
| 2019 | 652 | 467 | 185 |
| 2020 | 639 | 467 | 172 |

### 1.2 Age standardized suicide rates, total and in males and females Norway 2010-2020

|  | All | Male | Female |
| --- | --- | --- | --- |
| 2010 | 11.7 | 16.7 | 7.0 |
| 2011 | 12.5 | 18.4 | 6.9 |
| 2012 | 10.6 | 15.4 | 6.1 |
| 2013 | 11.2 | 15.5 | 7.1 |
| 2014 | 11.0 | 16.4 | 5.9 |
| 2015 | 11.8 | 16.1 | 7.7 |
| 2016 | 12.0 | 16.4 | 7.7 |
| 2017 | 11.5 | 15.7 | 7.4 |
| 2018 | 12.9 | 18.2 | 7.8 |
| 2019 | 12.4 | 17.8 | 7.1 |
| 2020 | 12.1 | 17.6 | 6.5 |

## 2.0 Geographic area

### 2.1 Number of suicides in Oslo area and rest of Norway, 2010-2020

|  | Oslo area | Norway excl.  Oslo area |
| --- | --- | --- |
| 2010 | 133 | 416 |
| 2011 | 135 | 466 |
| 2012 | 118 | 402 |
| 2013 | 125 | 430 |
| 2014 | 130 | 421 |
| 2015 | 140 | 456 |
| 2016 | 130 | 487 |
| 2017 | 135 | 460 |
| 2018 | 161 | 513 |
| 2019 | 177 | 475 |
| 2020 | 143 | 496 |

### 2.2 Age standardized suicide rates, in Oslo area and rest of Norway, 2010-2020.

|  | Oslo area | Norway excl.  Oslo area |
| --- | --- | --- |
| 2010 | 12.8 | 11.5 |
| 2011 | 12.1 | 12.7 |
| 2012 | 10.0 | 10.8 |
| 2013 | 11.2 | 11.4 |
| 2014 | 11.5 | 11.1 |
| 2015 | 11.6 | 11.9 |
| 2016 | 10.6 | 12.6 |
| 2017 | 10.8 | 11.7 |
| 2018 | 12.9 | 13.1 |
| 2019 | 13.7 | 12.0 |
| 2020 | 11.3 | 12.4 |

## 3.0 Age groups

### 3.1 Number of suicides in different age groups, Norway 2010-2020.

|  | 0-24 | 15-24 | 25-44 | 45-64 | 65- |
| --- | --- | --- | --- | --- | --- |
| 2010 | 74 | 73 | 181 | 197 | 97 |
| 2011 | 84 | 77 | 209 | 216 | 92 |
| 2012 | 70 | 68 | 181 | 190 | 79 |
| 2013 | 66 | 65 | 187 | 212 | 90 |
| 2014 | 58 | 55 | 187 | 207 | 99 |
| 2015 | 66 | 62 | 182 | 227 | 121 |
| 2016 | 72 | 70 | 218 | 231 | 96 |
| 2017 | 94 | 89 | 183 | 216 | 102 |
| 2018 | 88 | 83 | 216 | 252 | 118 |
| 2019 | 86 | 82 | 226 | 226 | 114 |
| 2020 | 77 | 72 | 221 | 208 | 133 |

### 3.2 Age standardized suicide rates in different age groups, Norway 2010-2020.

|  | 0-24 | 15-24 | 25-44 | 45-64 | 65- |
| --- | --- | --- | --- | --- | --- |
| 2010 | 4,8 | 11,5 | 13,4 | 15,8 | 13,2 |
| 2011 | 5,3 | 11,9 | 15,3 | 17,1 | 12,2 |
| 2012 | 4,4 | 10,3 | 13,1 | 14,9 | 10,1 |
| 2013 | 4,1 | 9,7 | 13,4 | 16,5 | 11,2 |
| 2014 | 3,6 | 8,2 | 13,3 | 15,9 | 12,0 |
| 2015 | 4,1 | 9,3 | 12,8 | 17,2 | 14,3 |
| 2016 | 4,5 | 10,5 | 15,3 | 17,3 | 11,1 |
| 2017 | 5,9 | 13,4 | 12,8 | 16,0 | 11,5 |
| 2018 | 5,5 | 12,5 | 15,0 | 18,4 | 13,0 |
| 2019 | 5,4 | 12,4 | 15,7 | 16,4 | 12,3 |
| 2020 | 4,9 | 11,0 | 15,2 | 14,9 | 13,9 |

## 4.0 Pandemic phases

### 4.1 January and February

### 4.1.1 Number of suicides in different age groups in January and February, Norway 2010-2020.

|  | All ages | 15-24 | 25-44 | 45-64 | 65- |
| --- | --- | --- | --- | --- | --- |
| 2010 | 88 | 6 | 38 | 33 | 11 |
| 2011 | 88 | 9 | 41 | 30 | 7 |
| 2012 | 90 | 11 | 32 | 35 | 11 |
| 2013 | 85 | 9 | 28 | 34 | 13 |
| 2014 | 91 | 11 | 31 | 35 | 14 |
| 2015 | 93 | 7 | 26 | 41 | 19 |
| 2016 | 107 | 13 | 33 | 42 | 19 |
| 2017 | 96 | 15 | 26 | 38 | 16 |
| 2018 | 96 | 9 | 35 | 32 | 17 |
| 2019 | 106 | 13 | 40 | 33 | 20 |
| 2020 | 118 | 14 | 41 | 41 | 21 |

### 4.2.2 Age standardized suicide rate in Norway in different age groups in January and February 2010-2020.

|  | All ages | 15-24 | 25-44 | 45-64 | 65- |
| --- | --- | --- | --- | --- | --- |
| 2010 | 7.3 | 0.9 | 2.8 | 2.6 | 1.5 |
| 2011 | 6.9 | 1.4 | 3.0 | 2.4 | 0.9 |
| 2012 | 7.2 | 1.7 | 2.3 | 2.8 | 1.4 |
| 2013 | 6.9 | 1.3 | 2.0 | 2.6 | 1.6 |
| 2014 | 7.3 | 1.6 | 2.2 | 2.7 | 1.7 |
| 2015 | 7.6 | 1.0 | 1.8 | 3.1 | 2.2 |
| 2016 | 8.5 | 1.9 | 2.3 | 3.1 | 2.2 |
| 2017 | 7.4 | 2.3 | 1.8 | 2.8 | 1.8 |
| 2018 | 7.4 | 1.4 | 2.4 | 2.3 | 1.9 |
| 2019 | 8.1 | 2.0 | 2.8 | 2.4 | 2.1 |
| 2020 | 8.9 | 2.1 | 2.8 | 2.9 | 2.2 |

### 4.2 March-May

### 4.2.1 Number of suicides in different age groups in March, April, and May, Norway 2010-2020.

|  | All ages | 15-24 | 25-44 | 45-64 | 65- |
| --- | --- | --- | --- | --- | --- |
| 2010 | 161 | 20 | 46 | 63 | 31 |
| 2011 | 156 | 18 | 42 | 67 | 26 |
| 2012 | 142 | 25 | 47 | 48 | 21 |
| 2013 | 159 | 14 | 65 | 51 | 29 |
| 2014 | 158 | 12 | 54 | 60 | 30 |
| 2015 | 144 | 22 | 36 | 54 | 31 |
| 2016 | 156 | 13 | 62 | 57 | 23 |
| 2017 | 162 | 33 | 48 | 56 | 23 |
| 2018 | 206 | 25 | 67 | 83 | 30 |
| 2019 | 166 | 27 | 55 | 59 | 25 |
| 2020 | 150 | 20 | 56 | 46 | 28 |

### 4.2.2 Age standardized suicide rate in Norway in different age groups in March, April, and May 2010-2020.

|  | All ages | 15-24 | 25-44 | 45-64 | 65- |
| --- | --- | --- | --- | --- | --- |
| 2010 | 14.0 | 3.1 | 3.4 | 5.1 | 4.2 |
| 2011 | 13.2 | 2.8 | 3.1 | 5.3 | 3.4 |
| 2012 | 11.5 | 3.8 | 3.4 | 3.8 | 2.7 |
| 2013 | 13.1 | 2.1 | 4.7 | 4.0 | 3.6 |
| 2014 | 13.0 | 1.8 | 3.8 | 4.6 | 3.6 |
| 2015 | 11.7 | 3.3 | 2.5 | 4.1 | 3.7 |
| 2016 | 12.1 | 1.9 | 4.3 | 4.3 | 2.7 |
| 2017 | 12.3 | 5.0 | 3.4 | 4.1 | 2.6 |
| 2018 | 15.7 | 3.8 | 4.7 | 6.1 | 3.3 |
| 2019 | 12.5 | 4.1 | 3.8 | 4.3 | 2.7 |
| 2020 | 11.4 | 3.0 | 3.9 | 3.3 | 2.9 |

### 4.3 June-September

### 4.3.1 Number of suicides in different age groups in June, July, August, and September, Norway 2010-2020.

|  | All ages | 15-24 | 25-44 | 45-64 | 65- |
| --- | --- | --- | --- | --- | --- |
| 2010 | 190 | 28 | 61 | 71 | 30 |
| 2011 | 222 | 33 | 70 | 80 | 38 |
| 2012 | 154 | 15 | 53 | 60 | 26 |
| 2013 | 182 | 21 | 52 | 81 | 28 |
| 2014 | 163 | 21 | 64 | 57 | 20 |
| 2015 | 212 | 20 | 67 | 80 | 43 |
| 2016 | 194 | 29 | 66 | 69 | 30 |
| 2017 | 189 | 24 | 62 | 64 | 39 |
| 2018 | 215 | 34 | 70 | 68 | 42 |
| 2019 | 219 | 28 | 74 | 75 | 42 |
| 2020 | 220 | 22 | 79 | 66 | 52 |

### 4.3.2 Age standardized suicide rate in Norway 2010-2020 in different age groups in June, July, August, and September.

|  | All ages | 15-24 | 25-44 | 45-64 | 65- |
| --- | --- | --- | --- | --- | --- |
| 2010 | 16.1 | 4.4 | 4.5 | 5.7 | 4.1 |
| 2011 | 18.7 | 5.1 | 5.1 | 6.3 | 5.0 |
| 2012 | 12.8 | 2.3 | 3.8 | 4.7 | 3.3 |
| 2013 | 14.8 | 3.1 | 3.7 | 6.3 | 3.5 |
| 2014 | 12.7 | 3.1 | 4.5 | 4.4 | 2.4 |
| 2015 | 17.2 | 3.0 | 4.7 | 6.1 | 5.1 |
| 2016 | 15.1 | 4.3 | 4.6 | 5.2 | 3.5 |
| 2017 | 15.0 | 3.6 | 4.3 | 4.7 | 4.4 |
| 2018 | 16.7 | 5.1 | 4.9 | 5.0 | 4.6 |
| 2019 | 16.8 | 4.2 | 5.1 | 5.4 | 4.5 |
| 2020 | 17.1 | 3.3 | 5.4 | 4.7 | 5.5 |

### 4.4 October-December

### 4.4.1 Number of suicides in different age groups in October, November, and December, Norway 2010-2020.

|  | All ages | 15-24 | 25-44 | 45-64 | 65- |
| --- | --- | --- | --- | --- | --- |
| 2010 | 110 | 19 | 36 | 30 | 25 |
| 2011 | 135 | 17 | 56 | 39 | 21 |
| 2012 | 134 | 17 | 49 | 47 | 21 |
| 2013 | 129 | 21 | 42 | 46 | 20 |
| 2014 | 139 | 11 | 38 | 55 | 35 |
| 2015 | 147 | 13 | 53 | 52 | 28 |
| 2016 | 160 | 15 | 57 | 63 | 24 |
| 2017 | 148 | 17 | 47 | 58 | 24 |
| 2018 | 157 | 15 | 44 | 69 | 29 |
| 2019 | 161 | 14 | 57 | 59 | 27 |
| 2020 | 151 | 16 | 45 | 55 | 32 |

### 4.4.2 Age standardized suicide rate in Norway 2010-2020 in different age groups in October, November, and December.

|  | All ages | 15-24 | 25-44 | 45-64 | 65- |
| --- | --- | --- | --- | --- | --- |
| 2010 | 9.7 | 3.0 | 2.7 | 2.4 | 3.4 |
| 2011 | 11.2 | 2.6 | 4.1 | 3.1 | 2.8 |
| 2012 | 11.0 | 2.6 | 3.5 | 3.7 | 2.7 |
| 2013 | 10.4 | 3.1 | 3.0 | 3.6 | 2.5 |
| 2014 | 11.9 | 1.6 | 2.7 | 4.2 | 4.3 |
| 2015 | 11.9 | 1.9 | 3.7 | 3.9 | 3.3 |
| 2016 | 12.5 | 2.2 | 4.0 | 4.7 | 2.8 |
| 2017 | 11.5 | 2.6 | 3.3 | 4.3 | 2.7 |
| 2018 | 12.2 | 2.3 | 3.1 | 5.0 | 3.2 |
| 2019 | 12.3 | 2.1 | 3.9 | 4.3 | 2.9 |
| 2020 | 11.6 | 2.4 | 3.1 | 4.0 | 3.4 |
